# Mechanistic *in vitro*–*in vivo* extrapolation (IVIVE) approach using a biomimetic *in vitro* system for the prediction of hepatic clearance

**DOI:** 10.1016/j.csbj.2025.05.036

**Published:** 2025-05-26

**Authors:** Seongwon Park, Sun-Sook Song, Woojin Jung, Hwi-yeol Yun, Soyoung Lee, Sun-Woong Kang, Jung-Woo Chae

**Affiliations:** a College of Pharmacy, Chungnam National University, 99, Daehak-ro, Yuseong-gu, Daejeon 34134, Republic of Korea; b Center for Biomimetic Research, Korea Institute of Toxicology, Daejeon 34114, Republic of Korea; c Senior Health Convergence Research Center, Chungnam National University, Daejeon 34134, Republic of Korea; d Department of Bio-AI convergence, Chungnam National University, 99, Daehak-ro, Yuseong-gu, Daejeon 34134, Republic of Korea; e School of Korea Institute of Toxicology, University of Science and Technology, Daejeon 34114, Republic of Korea

**Keywords:** 3 R principle, Biomimetic *in vitro* system, *In vitro*–*in vivo* extrapolation (IVIVE), Pharmacokinetic model, HepaRG cell

## Abstract

This study proposes a novel *in vitro*–*in vivo* extrapolation (IVIVE) strategy that integrates a patented biomimetic *in vitro* system with pharmacokinetic modeling to improve the prediction of *in vivo* drug kinetics. The system employed a single-well plate design with various sizes of mesh inserts to simultaneously assess drug diffusion and cellular metabolism. Drug diffusion patterns were quantified and modeled using a Weibull distribution to establish mathematical relationships between pore size and kinetic parameters. The model was extended to predict the metabolic phase with two drugs, the metabolic conversion of diclofenac into 4-hydroxydiclofenac and metabolic clearance of testosterone in HepaRG cells. The predicted *in vivo* hepatic clearance values derived from the *in vitro* model were consistent with those reported *in vivo*, validating the approach. This integrated strategy enhances IVIVE accuracy and supports the 3 R (replacement, reduction, and refinement) principle by reducing the reliance on animal testing. The findings demonstrate the potential of combining biomimetic systems and mathematical modeling as a reliable platform for pharmacokinetic prediction in drug development.

## Highlights

• A biomimetic system enables simultaneous analysis of drug diffusion and metabolism.
• Drug diffusion modeled by a Weibull equation reflected mesh pore size differences.
• *In vitro*–*in vivo* extrapolation (IVIVE) using HepaRG predicted hepatic clearance.
• Combining novel biomimetic *in vitro* system and modeling provides new IVIVE approach.
• IVIVE supports the “replacement, reduction, and refinement” principle.

## Graphical Abstract

**Figure:**
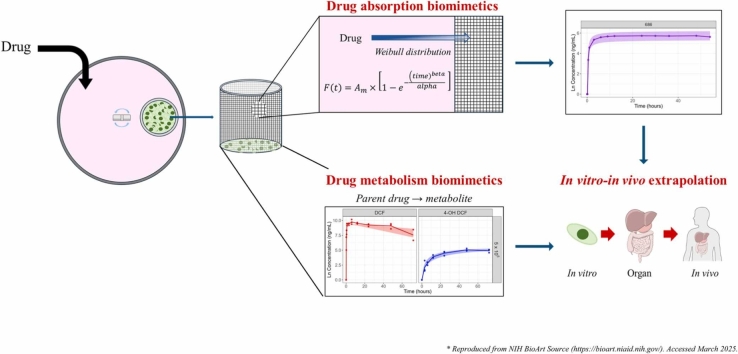

## 1 Introduction

In recent years, growing ethical awareness and scientific innovations have accelerated the search for alternatives to animal testing in drug development [1]. Central to this paradigm shift is the 3Rprinciple (replacement, reduction, and refinement), which promotes the minimization of animal use while maintaining scientific validity [2]. Regulatory agencies, such as the United States Food and Drug Administration (FDA) and European Medicines Agency (EMA), have actively supported these efforts by encouraging the incorporation of non-animal testing approaches, particularly in the fields of pharmacokinetics and toxicity evaluation [3], [4]. Moreover, regulatory requirements now support the introduction of *in vitro* testing methods to minimize or replace animal experiments in diverse areas such as ecotoxicity and food safety assessments [5]. These new methods show considerable potential for mimicking *in vivo* conditions without relying on animal models.

To address these demands, various *in vitro* experimental platforms have been developed, including Caco-2 monolayer cultures, microfluidic organ-on-a-chip systems, and three-dimensional (3D) cell culture models [6]. The Caco-2 model is widely used because of its simplicity, reproducibility, and established protocols for evaluating intestinal permeability [7]. However, as a static and metabolically-limited 2D system, it lacks the dynamic flow and enzymatic activity required to simulate complex *in vivo* pharmacokinetic processes, such as absorption and first-pass metabolism [8]. In contrast, microfluidic chip-based systems provide advanced physiological mimicry through dynamic fluid flow, mechanical stimuli, and multiorgan interactions [9]. These features allow for more realistic simulations of human organ functions [10]. Nevertheless, their broader application is often limited by their technical complexity, low throughput, and high operational costs, making them less feasible for routine use in early stage drug screening [11].

These limitations emphasize the need for *in vitro* systems that are not only physiologically relevant but also experimentally accessible, reproducible, and scalable [12]. Such platforms must be capable of accurately simulating drug behavior across key pharmacokinetic processes, including absorption, distribution, metabolism, and excretion (ADME), while enabling translation to *in vivo* scenarios through *in vitro–in vivo* extrapolation (IVIVE) [13]. From a pharmacokinetic perspective, the efficient use of *in vitro* data requires a model-based approach that can quantitatively bridge experimental observations with whole-body drug behavior [14]. Constructing physiologically-based pharmacokinetic (PBPK) models from *in vitro* parameters, such as permeability and metabolic rates, has shown considerable promise for simulating human pharmacokinetics [15]. Several studies have demonstrated that coupling data from hepatocytes or HepaRG cells with PBPK models can improve prediction accuracy while reducing the reliance on animal testing [16], [17].

In addition to their biological relevance, the development of mathematical models is essential to enhance the utility of *in vitro* systems [6]. Thus, recent efforts have focused on multi-chamber or sectioned *in vitro* platforms that incorporate elements such as porous membranes, multiphase, and modular configurations [18]. By integrating these advanced systems with mechanistic modeling, researchers can more accurately capture complex and time-dependent processes, such as nonlinear diffusion and multistep metabolism. This approach improves the physiological fidelity of IVIVE and supports the refinement of drug development pipelines through virtual trials, experimental design optimization, and minimized reliance on animal experiments [13], [19], [20].

Building on these advances, a novel biomimetic *in vitro* system was developed that combined a porous, mesh-based diffusion interface with liver-derived cells to simulate both drug transport and metabolism. By integrating experimental data with pharmacokinetic modeling based on Weibull distribution, a mechanistic IVIVE framework for predicting hepatic clearance was developed. The system was designed to be practical, scalable, and compatible with computational analysis, offering a promising alternative to existing models and supporting the development of animal-free, human-relevant pharmacokinetic prediction tools.

## 2 Methods

### 2.1 Biomimetic *in vitro* system

**Fig. 1:**
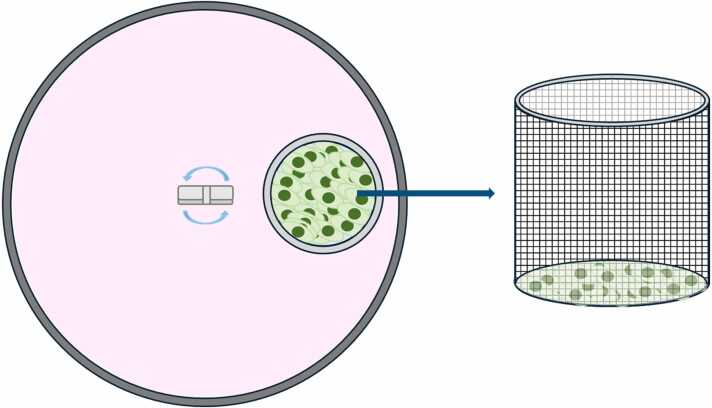
Schematic diagram of the biomimetic in vitro system. A single dish includes insert, and it is constructed with porous mesh. The porous insert is designed to influence the diffusion rate based on its pore size. A centrally positioned magnetic stirrer enables control of the diffusion rate. The system is designed to evaluate cellular responses to the drug by seeding cells within the insert, as desired by the investigator.

The structure and conditions of the biomimetic *in vitro* system (Korean Patent No. 10–2022–0153547) used in this study are shown in (Fig. 1) [21]. The system comprised an insert made of a porous mesh placed within a single-well plate. This design allowed the control of the drug diffusion rate using inserts with different pore sizes. The well plate was filled with medium, and a stirrer was placed inside the well plate to adjust the drug diffusion pattern according to the set rotational speed. In addition, different meshes were selected and applied depending on the study objective, and cell responses were evaluated using cells (e.g., liver-derived cells) cultured within the insert.

For this study, HepaRG cells, which are derived from a liver tumor of a female patient with hepatocarcinoma and hepatitis C infection, were cultured within the insert using Williams E medium (pH 7.03 – 7.43) supplemented with 10 % FBS, 1 % antibiotic-antimycotic solution, ITS-G supplement (2 μg/mL insulin, 1.1 μg/mL transferrin, 1.34 ng/mL sodium selenite), 1 % GlutaMAX-I, and 8.65 μM hydrocortisone hemisuccinate [22].

### 2.2 Mechanistic IVIVE for absorption phase

In this study, a model-based simulation of the drug diffusion-induced absorption process was constructed to explain the biomimetic *in vitro* system. To quantitatively describe the effect of different mesh pore sizes on the drug diffusion pattern, the Weibull distribution equation (see Eq. (1)) was applied [23]. This study omitted the lag time before the onset to simplify the formulation. We predicted the Weibull parameters that determined the drug transfer rate for each mesh size, such as A_m_ (maximum rate of drug release), α (scale factor), and β (shape factor). Regression analysis was used to derive the relationship between these parameters and the mesh size.

(1) $$F\left(t\right)=A_{m}\times \;\left[1-e^{-{\left(\frac{\mathrm{time}}{\alpha }\right)}^{\beta }}\;\right]$$

The absorption model was verified using observed data from triplicate measurements obtained over time after treating systems with various mesh sizes in media containing 50 μM rosiglitazone (357.43 g/mol). The mesh sizes used ranged from 125 to 686 mesh. For the 686 mesh, the structure was so dense that direct measurement using scanning electron microscopy (SEM) images was difficult; therefore, the pore size was estimated using regression analysis. This regression analysis assumed that the mesh used in the study had a uniform thread thickness and that the pores of the 686 mesh were square.

Based on the cumulative time-dependent rosiglitazone observations, Eq. (1) validated the parameters (A_m_, α, β) for each mesh. A regression analysis on the relationship between the estimated pore size and the Weibull parameters for each mesh was then conducted. Based on these results, a Weibull distribution equation for each parameter was derived that explained the diffusion kinetics between the media and the insert in relation to the pore size variation.

### 2.3 Mechanistic IVIVE for metabolism phase

By extending the previously-established Weibull distribution-based absorption model, a model was developed that predicted how the parent drug given to the medium diffused into the insert and was metabolized by cells to form metabolite or eliminated through other pathways, accounting for both metabolite accumulation and parent drug depletion over time. The original absorption model considers two compartments: media and insert. The extended model, which incorporated metabolite formation and kinetics, was developed with four compartments to comprehensively describe both parent drug disposition and metabolite dynamics (Supplementary Fig. 1). These compartments included a media compartment for the parent drug, an insert compartment for the parent drug, a media compartment for the metabolite, and an insert compartment for the metabolite.

The type and number of cells within the insert affected metabolic efficiency. To reflect this, a scaling factor was introduced that adjusted the metabolic reaction equation using cell counts. This scaling factor accounted for the fact that changes in cell count may not have resulted in a directly proportional change in metabolism. In addition, a different Weibull distribution equation was applied to explain the diffusion kinetics of the metabolites between the insert and media.

For model validation, we used data from two separate experiments utilizing different compounds: diclofenac and testosterone.

In the first experiment, HepaRG cells were seeded in the insert, and the media was treated with 40 μM diclofenac (296.15 g/mol). Diclofenac was added to the circulating medium and 100 μL samples were collected from the insert seeded with HepaRG cells at pre-determined time points post-diclofenac administration. Time-course measurements of the decrease in diclofenac concentration in the insert and the formation of its metabolite, 4-hydroxydiclofenac (312.15 g/mol), were taken. For the experiment, HepaRG cells were seeded into the insert at densities of 1, 2, and 5× 10⁵ cells per insert. The parent drug concentrations were measured at 0.17, 0.5, 1, 3, 6, 12, 24, 48, and 72 h, whereas the metabolite concentrations were measured at 3, 6, 12, 24, 48, and 72 h. All measurements were performed in triplicate under each condition.

In the second experiment, testosterone (288.42 g/mol) was used as the test compound at concentrations of 1, 5, and 20 μM. Similar to the diclofenac experiment, HepaRG cells were seeded into the insert, but at a fixed density of 5 × 10⁵ cells. Samples were collected and parent drug concentrations were measured at 1, 3, 6, 12, 24, 36, 48, and 72 h. No metabolite concentrations were measured in this experiment.

Both experiments were conducted using a 508 mesh; the Weibull parameters corresponding to the 508 mesh were derived using a previously-established regression analysis between pore size and Weibull distribution parameters. These fixed Weibull parameters for the absorption (diffusion) kinetics were used for model validation except for A_m_ parameter. Considering realistic experimental variability, a variability of 30 % was incorporated into the metabolism parameters within the model, and the metabolism phase model was validated accordingly.

To assess the robustness and reliability of the developed models, a bootstrap analysis was performed for both diclofenac and testosterone models (n = 500). For each iteration, the experimental data were resampled with replacement, and the model parameters were re-estimated. This procedure generated distributions of parameter values, from which the median and percent relative standard error (%RSE) were calculated for each model parameter. This bootstrap validation confirmed that both the diclofenac model and the testosterone model produced stable and reproducible parameter estimates, supporting the robustness of mechanistic metabolism model.

### 2.4 Evaluation of new IVIVE methods

Before performing IVIVE, a pharmacokinetic model was developed for the biomimetic *in vitro* system that precisely predicted the observed values of diclofenac, 4-hydroxydiclofenac and testosterone. Based on the metabolic parameters obtained using the model simulation, IVIVE was conducted to convert *in vitro* intrinsic clearance of HepaRG cells (*in vitro CL*_*int,HepaRG*_) into *in vivo* hepatic clearance (*in vivo CL*_*hepatic*_). First, the *in vitro CL*_*int,HepaRG*_ was converted to that of primary human hepatocytes (*in vitro CL*_*int,hepatocyte*_) by accounting for the difference in the metabolic capacity between the two cell types (Eq. (2)) [24]. Next, the *in vitro* intrinsic clearance for hepatocytes (*in vitro CL*_*int,hepatocyte*_) was scaled to the whole-liver level (*in vivo CL*_*int,liver*_) (Eq. (3)) [25] and then extrapolated to the whole-body level by assuming a 70 kg adult (*in vivo CL*_*int*_) (Eq. (4)) [26]. Finally, assuming a well-stirred model, we used hepatic blood flow (*Q*) and a fraction unbound in plasma (*f*_*u*_) to obtain the final *in vivo in vivo CL*_*hep*_ [27].

For precise IVIVE, the unbound fraction in incubation (*f*_*u,inc*_) for diclofenac and testosterone in hepatocyte was calculated using Eq. (6) [28] and incorporated into the hepatic clearance using Eq. (5) [29]. Specifically, *f*_*u,inc*_ was derived by considering the volume ratio of hepatocyte to the medium (*V*_*R*_) and log D. This value was then used to estimate the drug elimination rate in the liver so that the *in vitro* results were more closely approximated *in vivo*.

(2) $$\mathrm{Step}\;1:\mathrm{in vitro}\;CL_{\mathrm{int},\mathrm{hepatocyte}}=\mathrm{in vitro}\;CL_{\mathrm{int},\mathrm{HepaRG}}÷\mathrm{metabolic}\;\mathrm{activity}$$

(3) $$\mathrm{Step}\;2:\mathrm{in}\;\mathrm{viv}o\;CL_{\mathrm{int},\mathrm{liver}}=\mathrm{in}\;\mathrm{vitro}\;CL_{\mathrm{int},\mathrm{hepatocyte}}\times \mathrm{scaling}\;\mathrm{for}\;\mathrm{hepatocyte}\;\mathrm{to}\;\mathrm{liver}$$

(4) $$\mathrm{Step}\;3:\mathrm{in}\;\mathrm{vivo}\;CL_{\mathrm{int}}=\;in\;vivo\;CL_{\mathrm{int},\mathrm{liver}}\times \mathrm{scaling}\;\mathrm{for}\;\mathrm{liver}\;\mathrm{to}\;\mathrm{whole}\;\mathrm{body}\;\left(70\mathrm{kg}\right)$$

(5) $$\mathrm{Step}\;4:\mathrm{in}\;\mathrm{vivo}\;CL_{\mathrm{hepatic}}=\frac{Q\times f_{u}\times \frac{CL_{\mathrm{int}}}{f_{u,\mathrm{inc}}^{*}}\;}{Q+f_{u}\times \frac{CL_{\mathrm{int}}}{f_{u,\mathrm{inc}}^{*}}}$$

(6) $$f_{u,\mathrm{inc}}^{*}=\;\frac{1}{1+125\times V_{R}\times 10^{0.072\times \log P{/D}^{2}\;+0.067\times \log P/D-1.126}}$$

In this study, we applied several key assumptions during the experimental design, data analysis, and interpretation of results. These assumptions served as an important foundation for the development of our experimental methodology and IVIVE. Supplementary Table 1 summarizes the major assumptions categorized by their domains.

### 2.5 Software and data processing

Data analysis and model simulations were performed in an R (v 4.4.1) environment [30] using RStudio (v 2024.12.0 +467) as the development platform. Data were processed using the *dplyr* (v 1.1.4) and *tidyr* (v 1.3.1) packages. Simulation and validation of the drug diffusion model were performed using the *mrgsolve* (v 1.5.2) package, whereas the simulation and validation of the metabolic model employed the *nlmixr2* (v 3.0.1) package. Graphical visualization of the results was performed using the *ggplot2* package (v. 3.5.1).

## 3 Results

### 3.1 Regression analysis for pore size of the mesh

**Fig. 2:**
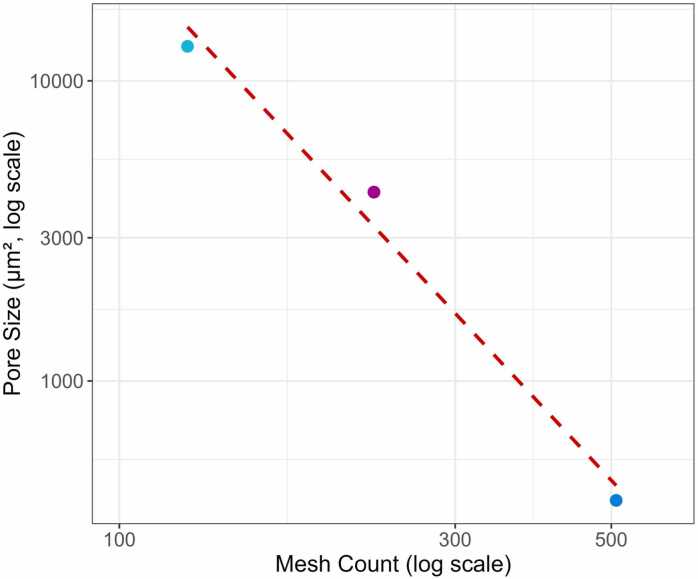
Regression analysis of mesh count versus pore size. Each dot represents the pore size corresponding to a specific mesh count (125, 230, and 508), and the red dashed line indicates the regression line. A high correlation was observed between the log₁₀-transformed mesh count and log₁₀-transformed pore size (R² = 0.984).

The meshes used in this study were classified into four types: 125, 230, 508, and 686. Actual measurements indicate pore sizes of 13,033 µm², 4259 µm², and 400 µm², for 125, 230, and 508 mesh, respectively. Regression analysis confirmed a strong correlation between mesh count and pore size (Table 1, R² = 0.984), and based on this, the pore size of the 686 mesh was estimated to be 211.05 µm² (Fig. 2).

**Table 1:** Pore size, Weibull distribution parameter values, and regression analysis results according to mesh count.

	**125 mesh**	**230 mesh**	**686 mesh**	**Regression equation**	**Description**
**Pore size (μm**^**2**^**)**	13033	4259	211	$\mathrm{Pore size}=10^{\left(-2.509\right)\times \log_{10}\left(\mathrm{Mesh}\right)+9.441}$	H (μm) x W (μm)
**A**_**m**_**(h**^**−1**^**)**	0.011	0.0024	0.00064	$A_{m}=0.00097\times e^{\left(0.00019\times \mathrm{Pore size}\right)}$	Maximum rate of drug released
**α (h**^**−1**^**)**	0.15	0.49	1.66	$\alpha =1.757\times e^{\left(-0.00029\times \mathrm{Pore size}\right)}$	Weibull parameter for scale
**β**	−12.1	−8.05	−2.61	$\beta =(-0.356)\times {\left(\mathrm{Pore size}\right)}^{0.373}$	Weibull parameter for shape

### 3.2 Validation of mechanistic absorption model for IVIVE

**Fig. 3:**
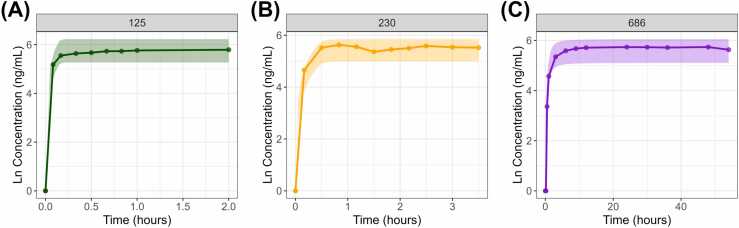
Simulation results of the mechanistic absorption model by mesh count. Panels (A), (B), and (C) display the time-dependent cumulative concentration profiles of rosiglitazone in the insert for 125, 230, and 686 mesh, respectively. Dots represent observed values, while the shaded area denotes the 90 % prediction interval.

**Fig. 4:**
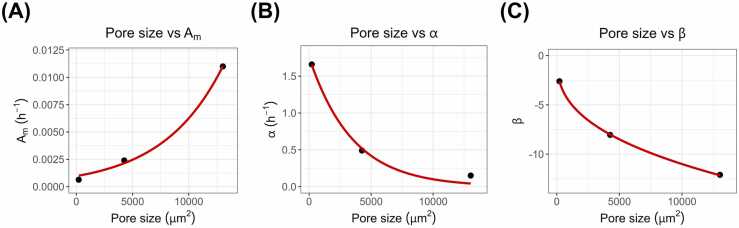
Regression analyses of pore size versus Weibull distribution parameters for the 125, 230, and 686 mesh in the mechanistic absorption model. Each data point represents a Weibull parameter calculated for a specific pore size from a given mesh count, while the red line denotes the fitted regression. (A) The A_m_ parameter displays a strong correlation with pore size based on exponential regression (R² = 0.997). (B) The α parameter shows a significant correlation under exponential decay regression (R² = 0.990). (C) The β parameter exhibits a high correlation with pore size according to ln-ln regression (R² = 1).

Reflecting on the decrease in pore size corresponding to the 125, 230, and 686 mesh sizes, a pharmacokinetic model was developed that captured the slower diffusion of the drug from the media into the insert and the delayed attainment of equilibrium. By applying the Weibull distribution equation to rosiglitazone, we performed 500 repeated simulations of the cumulative concentration profile of the drug in the insert. As the pore size decreased, the time required to reach the cumulative concentration equilibrium in the insert was delayed (Fig. 3). The Weibull parameters for each mesh (A_m_, α, and β) are summarized in Table 1. As the mesh count increased (pore size decreased), A_m_ exhibited a slight decrease, the scale parameter α decreased significantly, and the shape factor β tended to increase (Fig. 4). An exponential regression model of pore size and A_m_ indicated a strong correlation (R² = 0.997). The parameter, α, had a strong correlation with pore size in an exponential decay regression model (R² = 0.990), whereas a strong correlation in β was demonstrated using natural logarithm regression model (R² = 1). The goodness of fit for each Weibull distribution parameter was evaluated using residual metrics including root mean square error (RMSE), mean square error (MSE), mean absolute error (MAE), mean percentage error (MPE), and mean absolute percentage error (MAPE) (Supplementary Table 2). All regression models showed acceptable levels of error metrics, confirming the reliability of the derived regression equations for predicting Weibull parameters based on mesh pore size.

### 3.3 Validation of the mechanistic metabolism model for IVIVE

To explain the drug diffusion characteristics of the 508 mesh, the previously derived regression equations between mesh pore size and the Weibull distribution parameters were applied, yielding values of A_m_ = 0.001, α = 1.565, and β = -3.3. Subsequently, the model was extended by fixing the α and β parameters to account for differences in experimental conditions, such as stirring rate, while allowing the A_m_ parameter to estimate.

**Fig. 5:**
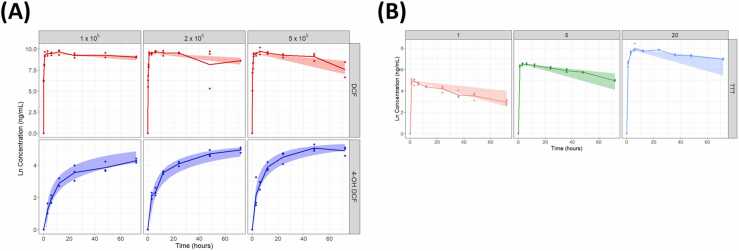
Simulation results of mechanistic metabolism model for (A) diclofenac and 4-hydroxydiclofenac and (B) testosterone. (A) The upper red data represent diclofenac, while the lower blue data correspond to the metabolite. The x-axis denotes time in hours and the y-axis denotes ln-transformed observed data. Dots indicate individual observations, the line represents the time-dependent mean of the observations, and the shaded region shows the 90 % prediction interval of the model predictions. (B) The three panels show simulation results at different testosterone concentrations: left panel (red) for 1 μM, middle panel (green) for 5 μM, and right panel (blue) for 20 μM. The x-axis denotes time in hours and the y-axis denotes ln-transformed observed data. Dots indicate individual observations, the line represents the time-dependent mean of the observations, and the shaded region shows the 90 % prediction interval of the model predictions.

The final estimated model parameters, %RSE values, and bootstrap results are summarized in Table 2. Using extended compartment models that integrated the absorption and metabolism phases, 500 simulations were performed for both diclofenac and testosterone (Supplementary Fig. 1). The simulation graph generated with estimated parameters is shown in Fig. 5, displaying the 90 % prediction interval produced by the variability of the metabolic parameters, demonstrating excellent concordance between the model predictions and the observed data.

**Table 2:** Final model parameters and bootstrap results of the mechanistic metabolism model.

Model parameter	**Unit**	Final estimate	**% RSE**	Bootstrap results		**Description**
				**Median**	**%RSE**	
**Diclofenac**						
vmax_p	h^−1^	0.0631	20	0.0635	8.82	Maximum rate of parent drug release
vmax_m	h^−1^	0.0299	1287	0.00118	221	Maximum rate of metabolite release
alpha_p	h^−1^	1.56 (FIX)	-	1.56 (FIX)	-	Weibull parameter for scale of parent drug
beta_p	-	−3.3 (FIX)	-	−3.3 (FIX)	-	Weibull parameter for shape of parent drug
meta	mL/min/cell	4.7 × 10^−6^	392	5.4 × 10^−6^	375	cellular metabolic clearance
portion	-	0.0154	55	0.0158	30.8	metabolic conversion fraction
scaling factor	-	0.643	31	0.627	16.2	scaling factor for cell count
**Testosterone**						
vmax	h^−1^	0.253	10.0	0.0253	2.23	Maximum rate of parent drug release
alpha	h^−1^	1.565 (FIX)	-	1.56 (FIX)	-	Weibull parameter for scale of parent drug
beta	-	−3.3 (FIX)	-	−3.3 (FIX)	-	Weibull parameter for shape of parent drug
meta	mL/min/cell	9.56 × 10^−6^	137.6	9.2 × 10^−6^	4.21	cellular metabolic clearance
scaling factor	-	0.589	0.9	0.592	0.607	scaling factor for cell count

The *‘meta’* parameter, representing clearance per HepaRG cell, was estimated at 4.7 × 10^-6^ mL/min/cell. Examination of model parameters indicated that the metabolic conversion rate of diclofenac to 4-hydroxydiclofenac was calculated at 1.5 %. The scaling factor for metabolic clearance relative to cell count was determined to be 0.643, effectively capturing the non-proportional metabolic kinetics of the parent drug with increasing cell numbers. Model estimation revealed high %RSE values for the metabolite *‘vmax’* parameter and *‘meta’* parameter (1287 % and 392 %, respectively), suggesting potential parameter uncertainty. However, bootstrap analysis provided more robust parameter estimates. The bootstrap median values were similar to the model estimates for all parameters except the metabolite *‘vmax’*, and importantly, showed substantially improved %RSE values across most parameters.

For the testosterone model, the *'meta'* parameter was estimated at 9.56 × 10^-6^ mL/min/cell. The scaling factor for cell count was determined to be 0.589, comparable to that observed in the diclofenac model. In the testosterone model, only the *'meta'* parameter showed a relatively high %RSE value (137.6 %) in the model estimation. Nevertheless, bootstrap analysis confirmed the robustness of the final parameter estimates. The code for the final pharmacokinetic model for the biomimetic *in vitro* system is provided in the Supplementary data.

**Table 3:** Physiological and drug-specific parameters used for IVIVE from HepaRG cells to human hepatic clearance.

Parameter	Value	Unit	Description	Reference
HPGL scaling factor	99	10⁶ cells/g liver	Hepatocytes per gram of liver	[25]
Liver scaling factor	25.7	g liver/kg body weight	Liver weight scaling for 70 kg adult	[26]
Q	20.7	mL/min/kg	Hepatic blood flow	[26]
**Diclofenac**				
*CL*_*int,HepaRG*_	0.078	mL/min/10⁶ cells	*In vitro* intrinsic clearance for HepaRG cells	Model simulation
Metabolic activity ratio	0.3	-	HepaRG cells metabolic activity relative to hepatocytes	[24]
*f*_*u*_	0.005	-	Unbound fraction in blood	[31]
*f*_*u,inc*_	0.963	-	Unbound fraction in HepaRG cells incubation	[28], [38]
**Testosterone**				
*CL*_*int,HepaRG*_	0.159	mL/min/10⁶ cells	*In vitro* intrinsic clearance for HepaRG cells	Model simulation
Metabolic activity ratio	0.7	-	HepaRG cells metabolic activity relative to hepatocytes	[24]
*f*_*u*_	0.01 – 0.04	-	Unbound fraction in blood	[32]
*f*_*u,inc*_	0.872	-	Unbound fraction in HepaRG cells incubation	[28], [38]

### 3.4 Application of a mechanistic model to IVIVE

The metabolic parameters obtained from the model were converted into appropriate units to derive the *in vitro* intrinsic clearance for both diclofenac and testosterone. The *in vitro CL*_*int,HepaRG*_ was calculated as 0.078 mL/min/10⁶ cells for diclofenac and 0.159 mL/min/10⁶ cells for testosterone from mechanistic metabolism model. These values were then extrapolated to hepatocytes by adjusting for the relative metabolic activity of HepaRG cells (30 % for diclofenac and 70 % for testosterone) [24], yielding *in vitro CL*_*int,hepatocyte*_ of 0.261 and 0.228 mL/min/10⁶ cells, respectively.

Both compounds were scaled to whole body clearance using standard physiological parameters: HPGL factor of 99 × 10⁶ hepatocytes/g liver [25] and liver weight of 25.7 g/kg for a 70 kg adult [26]. Using IVIVE approach, the *in vivo CL*_*int*_ was determined to be 664.35 mL/min/kg for diclofenac and 579.13 mL/min/kg for testosterone.

To calculate *in vivo CL*_*hepatic*_ using the well-stirred model, compound-specific parameters were incorporated with hepatic blood flow of 20.7 mL/min/kg [26]. For diclofenac, with *f*_*u*_ = 0.5 % [31] and *f*_*u,inc*_ = 0.963, the *in vivo CL*_*hepatic*_ value was 2.91 mL/min/kg. For testosterone, with *f*_*u*_ = 1 – 4 % [32] and *f*_*u,inc*_ = 0.872, the *in vivo CL*_*hepatic*_ ranged from 5.03 – 11.63 mL/min/kg. All parameters and calculations are summarized in Tables 3–4 following (2), (3), (4), (5), (6):

**Table 4:** IVIVE results from metabolic parameter of final model to *in vivo* hepatic clearance.

IVIVE	Description	Value	Unit	Equation
**Diclofenac**				
*meta*_*HepaRG*_	metabolism parameter from HepaRG cell	4.7 × 10^−6^	1/h/cell	
*in vitro CL*_*int,HepaRG*_	*in vitro* intrinsic clearance for HepaRG cell	0.0783	mL/min/10^6^ cell	
*in vitro CL*_*int,hepatocyte*_	*in vitro* intrinsic clearance for hepatocyte	0.261	mL/min/10^6^ cell	Eq. 2
*in vivo CL*_*int,liver*_	scaling for hepatocyte to liver weight	25.85	mL/min/g liver	Eq. 3
*in vivo CL*_*int*_	scaling for liver weight to body weight (70 kg) (= *in vivo* intrinsic clearance)	664.35	mL/min/kg	Eq. 4
*in vivo CL*_*hepatic*_	*in vivo* hepatic clearance	2.91	mL/min/kg	Eq. 5
**Testosterone**				
*meta*_*HepaRG*_	metabolism parameter from HepaRG cell	9.56 × 10^−6^	1/h/cell	
*in vitro CL*_*int,HepaRG*_	*in vitro* intrinsic clearance for HepaRG cell	0.1593	mL/min/10^6^ cell	
*in vitro CL*_*int,hepatocyte*_	*in vitro* intrinsic clearance for hepatocyte	0.228	mL/min/10^6^ cell	Eq. 2
*in vivo CL*_*int,liver*_	scaling for hepatocyte to liver weight	22.53	mL/min/g liver	Eq. 3
*in vivo CL*_*int*_	scaling for liver weight to body weight (70 kg) (= *in vivo* intrinsic clearance)	579.13	mL/min/kg	Eq. 4
*in vivo CL*_*hepatic*_	*in vivo* hepatic clearance (1 – 4 % for fraction unbound)	5.03 – 11.63	mL/min/kg	Eq. 5

## 4 Discussion

This study presents a novel *in vitro–in vivo* extrapolation (IVIVE) framework that integrates a biomimetic *in vitro* system with a mechanistic pharmacokinetic model. The system was designed to simultaneously evaluate drug diffusion and metabolism using a porous mesh insert and HepaRG cells, and was mathematically modeled using a Weibull distribution-based kinetic approach to simulate drug behavior across compartments. By applying this system to the well-characterized reference compound, diclofenac, we demonstrated its capacity to generate *in vitro*-derived metabolic parameters that closely approximate *in vivo* hepatic clearance.

Conventional *in vitro* systems, such as monolayer cultures or simple hepatocyte assays, often fail to capture the dynamic interplay between drug transport and metabolism, and existing diffusion models rarely account for the physical characteristics of the culture environment. To address these limitations, the present system incorporated adjustable mesh pore sizes that enable the precise control of drug diffusion rates, thereby allowing more accurate mimicry of *in vivo* concentration gradients. The results of this study further highlighted how drug diffusion characteristics strongly affect pharmacokinetic profiles *in vitro*. By applying a mesh-regulated diffusion framework, physical transport could be decoupled from the metabolic capacity, thereby providing a more refined analysis of each component. This approach is particularly advantageous for evaluating compounds with rate-limiting absorption or metabolism, and may aid in designing more physiologically-relevant *in vitro* models for other ADME processes. Moreover, the incorporation of Weibull-based modeling enhanced the predictive power of the system by mathematically capturing the nonlinear diffusion kinetics as a function of the mesh structure.

To demonstrate the generalizability of our mechanistic modeling approach, we validated the methodology using two distinct compounds with different pharmacokinetic properties: diclofenac and testosterone. These compounds were strategically selected as they represent FDA-designated probe substrates for two major hepatic cytochrome P450 enzymes. Diclofenac serves as a probe substrate for cytochrome P450 2C9 (CYP2C9) while testosterone is used for cytochrome P450 3A4 (CYP3A4). Together, these enzymes account for a significant portion of drug metabolism in humans. While both compounds undergo hepatic metabolism, they exhibit markedly different physicochemical characteristics. Diclofenac is an acidic non-steroidal anti-inflammatory drug with relatively high protein binding (>99 %), whereas testosterone is a neutral steroid hormone with moderate binding (96 – 99 %). Our models successfully captured the distinct metabolic profiles of both compounds. The diclofenac model incorporated metabolite formation (4-hydroxydiclofenac) while the testosterone model focused on parent drug disappearance. The IVIVE calculations yielded hepatic clearance values of 2.91 mL/min/kg for diclofenac and 5.03 – 11.63 mL/min/kg for testosterone, which align well with reported *in vivo* values. This dual-compound validation strengthens confidence in the broader applicability of *in vitro* biomimetic system for predicting human hepatic clearance across diverse drug classes.

The use of the Weibull distribution equation to describe drug diffusion in *in vitro* biomimetic system was driven by the empirical observation of drug transfer kinetics between the insert and media chambers. The experimental data revealed a characteristic temporal profile where drug movement exhibited an initial rapid phase followed by gradual deceleration, eventually approaching zero as equilibrium was reached. This diminishing transfer rate pattern could not be adequately captured by conventional zero- or first-order kinetic models, which assume constant or proportionally constant rates. The Michaelis-Menten equation was deemed theoretically unsuitable for describing passive diffusion processes, while transit compartment models, although capable of describing parent drug kinetics, provided insufficient explanation for metabolite behavior as demonstrated in our preliminary studies. The Weibull distribution equation successfully reproduced the observed time-dependent decay without imposing mechanistic assumptions inconsistent with passive diffusion, the predominant mechanism in our system. Rather than representing the underlying physics of diffusion, the Weibull function serves as an empirical descriptor that effectively captures the specific pattern of transfer rate decay over time, making it a suitable mathematical tool for our modeling purposes.

The concentrations selected for diclofenac (40 µM) and testosterone (1, 5, 20 µM) were strategically determined based on toxicological and analytical considerations. For diclofenac, the 40 µM concentration exceeds typical systemic *C*_*max*_ values (4.7 – 10.1 µM) following oral administration [33], but was chosen to ensure effective metabolite detection and profiling while remaining well below the acute cytotoxicity threshold in HepaRG cells (EC_50_: 262 µM) [34]. This concentration allows for robust production of primary and secondary metabolites, including 4-hydroxydiclofenac and glucuronide conjugates, facilitating comprehensive metabolic assessment. Additionally, this level may reflect elevated hepatic exposure during portal first-pass metabolism and enterohepatic recirculation [35]. Similarly, testosterone concentrations of 1 – 20 µM were selected to enable effective detection of CYP3A4-mediated metabolites. While these levels exceed physiological concentrations (0.009 – 0.032 µM in males) [36], they fall within the range successfully used in previous HepaRG studies, which have employed concentrations up to 50 µM for metabolic assessments without inducing cytotoxicity [37]. These concentrations have demonstrated effective testosterone metabolism in HepaRG cells, with detectable formation of 6β-hydroxytestosterone and other metabolites. Importantly, the selected concentrations account for fundamental differences between *in vitro* and *in vivo* systems, particularly regarding protein binding and tissue distribution factors. In *in vivo* conditions, plasma protein binding and hepatic protein concentrations significantly influence free drug availability for metabolism, whereas *in vitro* cellular systems lack these complex distribution mechanisms. The use of both compounds at these carefully selected concentrations validates our system's capability to evaluate drug metabolism across two major hepatic CYP isoforms (CYP2C9 and CYP3A4) under conditions that balance analytical feasibility with cellular viability while accounting for system-specific distribution differences.

An important finding of this study was that metabolic clearance did not increase proportionally with increasing cell number. Although the absolute amount of metabolite increased with higher seeding densities, the per-cell clearance rate was 1.56-fold higher in 2 × 10⁵ cells and 2.81-fold higher in 5 × 10⁵ cells compared to 1 × 10⁵ cells. This nonlinear relationship indicates that factors beyond cell numbers may influence metabolic outcomes. This phenomenon can be attributed to differential proliferative behavior that depends on the initial seeding density. Cells seeded at lower densities likely had sufficient space and time to proliferate during the 3-day drug exposure period, effectively increasing the number of metabolically-active cells. In contrast, cells seeded at higher densities may have reached confluence earlier and experienced contact inhibition due to spatial limitations, thereby restricting further growth. These differences in growth dynamics may explain the nonproportional increase in metabolism relative to the number of cells.

According to the literature, *in vivo* hepatic and total body clearances of diclofenac are 4 mL/min/kg and 28 L/h, respectively [38], [39]. In the current study, the metabolism of diclofenac was evaluated using HepaRG cells. Immortalized HepaRG cells exhibited only approximately 30 % of the metabolic capacity of primary human hepatocytes owing to the reduced expression of key enzymes, such as cytochrome P450. When the *in vitro* intrinsic clearance for HepaRG cell of 0.0783 mL/min/10^6^ cells obtained from the current study was adjusted to account for the diminished metabolic capacity of HepaRG cells, the corrected *in vitro* intrinsic clearance for hepatocyte was approximately 0.261 mL/min/10^6^ cells.

When referring to the previously reported metabolic clearance rate of testosterone, values range from 304 to 516 L/day/m² or 812–1272 L/day [40], [41]. Converting these values to the same units used in our study, assuming a standard body surface area of 1.81 m² for normal-weight adults (70 kg for males and 60 kg for females), yields a range of 9.17 ± 1.60–15.84 ± 2.09 L/day/kg [42]. This closely aligns with the *in vivo* hepatic clearance of testosterone derived in our study, which ranges from 5.03 to 11.63 mL/min/kg (equivalent to 7.24 – 16.75 L/day/kg). These findings confirm that the values predicted through IVIVE from our pharmacokinetic model, which was constructed based on observations from a biomimetic *in vitro* system, demonstrate reasonable agreement with previously reported values in literature.

The final parameters from the metabolism model indicated that the metabolic fraction from diclofenac to 4-hydroxydiclofenac was estimated as 1.5 %. In humans, approximately 50 % of diclofenac is metabolized to 4-hydroxydiclofenac, while 10–20 % is excreted as an acyl glucuronide conjugate [43]. However, unlike typical hepatocytes, HepaRG cells predominantly follow the acyl glucuronide conjugate pathway with a relatively minor hydroxylation pathway [44]. Although quantitative studies on diclofenac to 4-hydroxydiclofenac metabolism in HepaRG cells are limited, considering the inherent variability of the *in vitro* assay system (e.g., cell passage differences), the results derived from our model appear to be realistic.

In the present study, we constructed a one-compartment model, setting the central compartment clearance to the *in vivo* hepatic clearance derived from our research and applying the apparent volume of distribution (*V*_*d*_ for a 70 kg individual) of diclofenac reported in the literature as the central compartment [33]. This model was then compared with reported single-dose PK study results for diclofenac (Supplementary Table 3) [45], [46]. For the absorption phase, we applied the Weibull distribution parameters obtained from our in vitro experiments. When comparing the pharmacokinetic parameter ranges calculated by applying 30 % variability to the central compartment clearance with clinical literature values, *C*_*max*_ and *AUC* were predicted to be slightly lower than clinical results. However, the predicted half-life (T_*1/2*_) was remarkably similar to the *in vivo* half-life of diclofenac observed in clinical trials. Drug absorption in actual biological systems involves significantly more complex mechanisms than our *in vitro* experimental design, making it difficult for this model to fully reflect the absorption process. Nevertheless, the high concordance between the half-life, which represents drug elimination characteristics, and clinical trial results effectively demonstrates the capability of our proposed methodology to predict drug elimination processes accurately.

However, this study had several limitations. First, using an immortalized cell line such as HepaRG introduces potential discrepancies in metabolic ability and enzyme expression that require adjustment for direct comparisons to *in vivo* conditions. In this study, we applied several assumptions in cellular experiments and modeling, particularly regarding metabolic activity which directly affects the accuracy of IVIVE results. Our assumptions regarding HepaRG cell metabolic activity were based on studies by Kanebratt et al. [24], where we assumed CYP3A4 activity in HepaRG cells to be 70 % of that in primary hepatocytes for IVIVE calculations. However, the metabolic activity difference (70 %) for testosterone used in this study was based on reported differences for midazolam metabolism. According to Kenworthy et al. [47], these two substrates bind to different active sites of CYP3A4, and such substrate specificity can influence actual metabolic activity. Although we were aware of these substrate-specific differences, we utilized this value as the primary objective of this study was to propose a novel IVIVE methodology. Nevertheless, this study demonstrates a novel IVIVE approach that contributes to advancing methodological frameworks for reducing reliance on animal testing. Second, while this study successfully validated the proposed IVIVE methodology using two representative probe substrates for CYP isoforms and demonstrated comparable clearance values to previously reported data, the limited number of test compounds restricts the generalization of these findings. Nevertheless, the successful validation of this novel approach demonstrates its potential utility for IVIVE applications.

In this study, we applied these metabolic activity correction factors to perform IVIVE based on primary hepatocytes rather than direct IVIVE using HepaRG cell metabolic capacity. As our study primarily focuses on proposing a new IVIVE methodology, we suggest that future research should clearly establish substrate-specific metabolic activity differences between the specific cellular model chosen by researchers and primary hepatocytes for more accurate IVIVE. This will be a crucial step in improving the accuracy of cell-based drug metabolism studies and enhancing the predictive power from *in vitro* to *in vivo*.

Despite these limitations, this study demonstrated the utility of combining biomimetic design with mathematical modeling for pharmacokinetic prediction. By integrating the biomimetic *in vitro* system with mechanistic equations, this flexible, scalable, and animal-free method effectively simulated *in vivo* behavior, while remaining compatible with model optimization, and virtual simulation strategies. Model-based simulation results empirically illustrate the potential of the refined IVIVE using human-relevant *in vitro* assays. This framework quantitatively reproduces key pharmacokinetic parameters by integrating drug diffusion and metabolic kinetics with cellular characteristics and mesh properties, systematically accounting for individual cell metabolic abilities, diffusion rates, and scaling factors. This finding suggests that IVIVE can closely approximate human conditions without relying on animal testing. Furthermore, the adjustable mesh characteristics of our system could potentially be extended to study drug permeability across different biological barriers, including intestinal absorption models, though such applications require systematic evaluation with drugs of varying physicochemical properties. Moreover, if further developed, this approach is expected to enhance the predictive accuracy of *in vitro* assays for diverse drugs and cell lines, ultimately evolving into a sophisticated pharmacokinetic prediction system that can effectively replace animal experiments for drug development and safety evaluations.

To enhance the accuracy of IVIVE predictions, future models may benefit from incorporating dynamic variables, such as proliferation rates, spatial constraints, and cell viability over time. Integrating these biological parameters could yield more precise clearance estimates and improve the scalability of *in vitro* models to physiologically-relevant conditions.

In summary, this study highlighted the importance of incorporating the physical microenvironment, mathematical modeling, and biological dynamics to enhance the accuracy of IVIVE. By offering a mechanistically-informed and practical alternative to traditional models, the biomimetic mesh-based system has potential to advance 3R-compliant drug development and personalized pharmacokinetic assessments, reducing the reliance on animal testing and increasing clinical relevance.

## 5 Conclusion

We demonstrated that a pharmacokinetic model based on a patented biomimetic *in vitro* system could accurately predict the complex absorption and metabolic kinetics observed *in vivo* and can be successfully applied to IVIVE. Our integrated approach produced promising IVIVE outcomes and showed potential in approximating human *in vivo* conditions. Furthermore, this model-based IVIVE strategy aligns with the international 3 R guidelines and supports the transition toward reducing animal testing. We anticipate that extending this approach to a broader range of drugs and cellular models will pave the way for a robust pharmacokinetic prediction platform capable of effectively complementing or even replacing animal studies.

## Code availability

The model code used in this study is available at [https://github.com/seongwonpark0122/New-IVIVE-approach]. An interactive R Shiny-based web application for model simulation can be accessed at [https://seongwonpark0122.shinyapps.io/New-IVIVE-approach/].

## CRediT authorship contribution statement

**Woojin Jung:** Writing – review & editing, Validation, Methodology, Investigation, Formal analysis, Data curation, Conceptualization. **Sun-Sook Song:** Writing – review & editing, Writing – original draft, Visualization, Validation, Resources, Project administration, Methodology, Investigation, Formal analysis, Data curation, Conceptualization. **Seongwon Park:** Writing – review & editing, Writing – original draft, Visualization, Validation, Project administration, Methodology, Investigation, Formal analysis, Data curation, Conceptualization. **Soyoung Lee:** Writing – review & editing, Writing – original draft, Visualization, Validation, Supervision, Methodology, Investigation, Formal analysis, Data curation, Conceptualization. **Hwi-yeol Yun:** Writing – review & editing, Writing – original draft, Visualization, Validation, Supervision, Methodology, Investigation, Formal analysis, Data curation, Conceptualization. **Jung-Woo Chae:** Writing – review & editing, Writing – original draft, Visualization, Validation, Supervision, Resources, Investigation, Formal analysis, Data curation, Conceptualization. **Sun-Woong Kang:** Writing – review & editing, Writing – original draft, Visualization, Validation, Supervision, Resources, Methodology, Investigation, Formal analysis, Data curation, Conceptualization.

## Funding

This study was supported by Chungnam National University, Institute of Information & communications Technology Planning Evaluation (IITP) grant funded by the Korea government (10.13039/501100014188MSIT) (No. RS-2022–00155857, Artificial Intelligence Convergence Innovation Human Resources Development (Chungnam National University)) and National Research Foundation of Korea (NRF) grant funded by the Korea government (MSIT; No. RS-2023–00278597, RS-2022-NR069643, RS-2022-NR070856), the Korea Environmental Industry & Technology Institute (10.13039/501100003654KEITI) through Core Technology Development Project for Environmental Diseases Prevention and Management (RS-2021-KE001333), funded by the Korea Ministry of Environment (MOE), a grant of the Korea Machine Learning Ledger Orchestration for Drug Discovery Project (K-MELLODDY), funded by the Ministry of Health & Welfare and Ministry of Science and ICT, Republic of Korea (grant number: RS-2024–00460694), the Korea Institute of Toxicology (10.13039/100009133KIT) Research Program (no. 2710008763, KK-2401–01), Korea Health Technology R&D Project through the Korea Health Industry Development Institute (10.13039/501100003710KHIDI) grant funded by the Ministry of Health & Welfare, Republic of Korea (grant number: RS-2024–00336984), supported by the Ministry of Trade, Industry, and Energy (MOTIE), Korea, under the “Infrastructure program for industrial innovation” supervised by the Korea Institute for Advancement of Technology (10.13039/501100003661KIAT)(RS-2024–00434342), and a grant (RS-2024–00331852) from the 10.13039/501100003569Ministry of Food and Drug Safety.

## Declaration of Competing Interest

The authors declare that they have no known competing financial interests or personal relationships that could have appeared to influence the work reported in this paper.

## Footnotes

Appendix A Supplementary data associated with this article can be found in the online version at doi:10.1016/j.csbj.2025.05.036.
