# Supplementary material for: Mechanistic in vitro–in vivo extrapolation (IVIVE) approach using a biomimetic in vitro system for the prediction of hepatic clearance
Source: Comput Struct Biotechnol J. 2025 May 26;27:2424–33. doi: 10.1016/j.csbj.2025.05.036 (PMC12173689; doi:10.1016/j.csbj.2025.05.036)
Supplement: Supplementary file 1 — Supplementary material [file mmc1.docx]

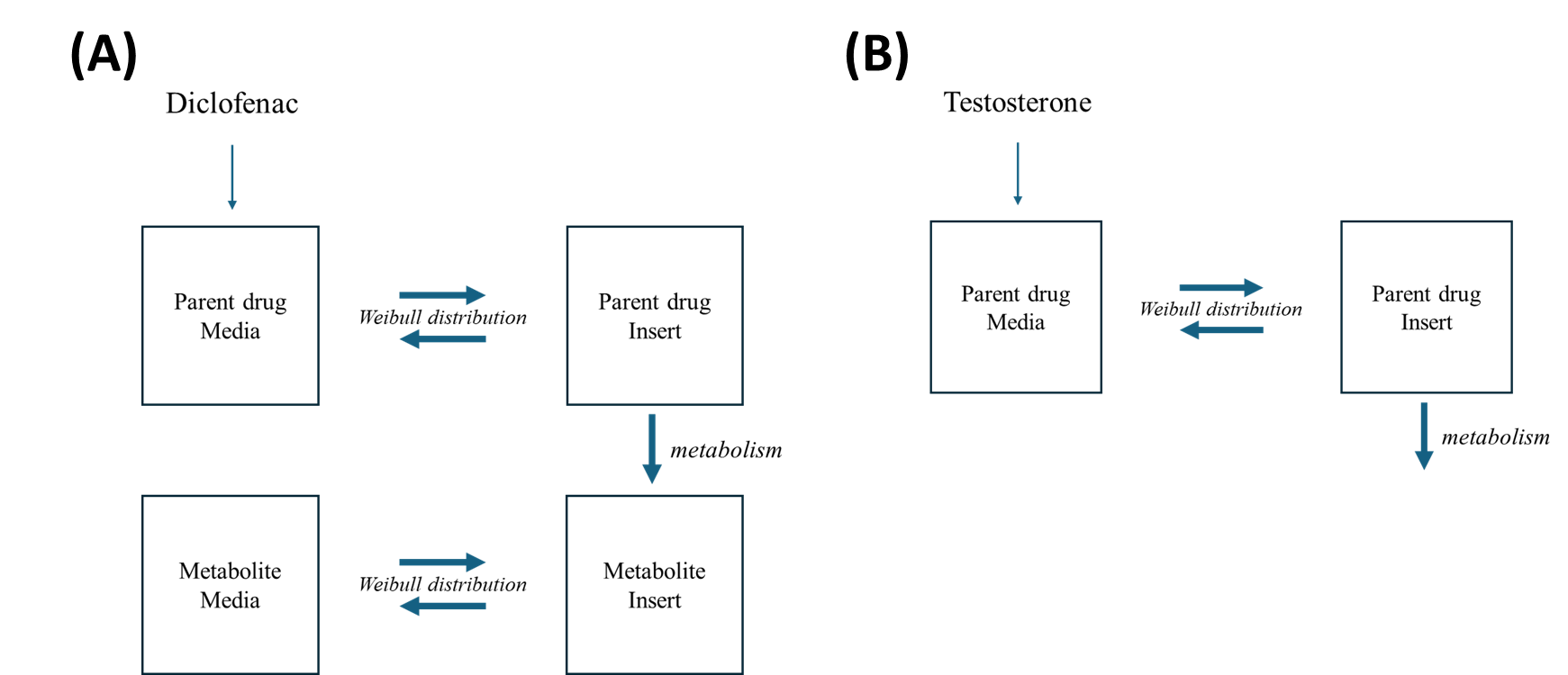


**Supplementary Fig. 1**. Scheme of pharmacokinetic model for biomimetic *in vitro* system. (A) Four‐compartment model for diclofenac incorporating both parent drug and metabolite (4‐hydroxydiclofenac) kinetics. The model describes bidirectional Weibull distribution‐based diffusion between media and insert compartments for both parent drug and metabolite, with metabolic conversion occurring within the insert compartment. (B) Two‐compartment model for testosterone focusing on parent drug kinetics only. Similar to the diclofenac model, it employs Weibull distribution‐based diffusion for drug diffusion between compartments.

**Supplementary Table. 1.**

Summary of key assumptions applied in this study.

| **Category** | **Assumption** | **Description** |
| --- | --- | --- |
| **Cell culture** |  |  |
|  | HepaRG cells demonstrate uniform growth during cultivation. | Uniformity in cell density and morphological characteristics was ensured through standardized culture conditions. |
|  | Cells are homogeneously distributed within the culture chamber. | Ensures consistency in drug exposure and metabolic activity, minimizing errors due to local heterogeneities. |
| **Cell metabolism** |  |  |
|  | HepaRG cells exhibit 30% metabolic activity for diclofenac compared to primary hepatocytes. | Based on studies by K. P. Kanebratt et al. (2008) [24], reflecting that CYP2C9 activity in HepaRG cells is approximately 30% of primary hepatocytes. |
|  | HepaRG cells exhibit 70% metabolic activity for testosterone compared to primary hepatocytes. | Based on studies by K. P. Kanebratt et al. (2008) [24], reflecting that CYP3A4 activity in HepaRG cells is approximately 70% of primary hepatocytes. |
|  | Enzyme activity remains stable throughout the experimental period. | Assumes no significant changes in CYP enzyme expression levels during the experimental period. |
| **Mesh structure** |  |  |
|  | For 686 mesh pore size calculations, mesh strands have uniform thickness and pores are square‐shaped. | Applied simplified geometric modeling based on manufacturer specifications and microscopic measurements. |
|  | Bidirectional transport across the mesh occurs at identical rates. | Following Fick's law of diffusion, a fundamental principle of passive diffusion, assuming equal diffusion rates when concentration gradients are identical. |
| **Drug binding** |  |  |
|  | Drug binding to proteins reaches equilibrium rapidly relative to metabolic timescale. | Protein binding equilibrium typically occurs within seconds to minutes, thus maintained throughout metabolism experiments lasting several hours. |
|  | Protein binding characteristics in the medium follow similar mechanisms to in vivo plasma protein binding. | Despite differences in protein types, relationships between fu,inc and fu were applied based on similarities in binding principles. |
| **Pharmacokinetic modeling** |  |  |
|  | The liver behaves as a well-stirred compartment for IVIVE calculations. | Applied the well-stirred model for predicting hepatic clearance, assuming uniform drug distribution within the liver. |
|  | Physiological parameters are based on a standard 70kg adult male. | Scaling factors such as liver weight, hepatocellularity, and blood flow were applied based on standard adult male references, without considering individual variations. |

**Supplementary Table 2.**

Regression model fit metrics for Weibull distribution parameters (A_m_, α, and β).

|  | **Regression equation** | **RMSE** | **MSE** | **MAE** | **MPE** | **MAPE** |
| --- | --- | --- | --- | --- | --- | --- |
| **A_m_ (h^-1^)** | $A_{m}=0.00097\times e^{\left( 0.00019\times Pore size \right)}$ | 0.0646 | 0.0042 | 0.0473 | -22.30 | 26.06 |
| **α (h^-1^)** | $\alpha=1.757\times e^{(-0.00029\times Pore size)}$ | 0.0367 | 0.0013 | 0.0311 | 0.00 | 0.35 |
| **β** | $\beta=(-0.356)\times\left( Pore size \right)^{0.373}$ | 0.0003 | 7×10^-8^ | 0.0002 | 15.75 | 22.80 |

**Supplementary Table 3.**

Comparison of predicted pharmacokinetic parameters for diclofenac (50 mg) with published clinical data.

| **Reference** | **Dose (mg)** | **C_max_ (ng/mL)** | **AUC_last_ (ng×h/mL)** | **AUC_inf_ (ng×h/mL)** | **T_1/2_ (h)** | **T_max_ (h)** |
| --- | --- | --- | --- | --- | --- | --- |
| **This study** | 50 | 141.97 – 197.61 | 377.91 – 701.73 | 378.31 – 702.37 | 1.44 – 2.72 | 1.4 – 1.6 |
| **Michael Lissy et al (2010)** | 50 | 1168±657 | 1131±391 | 1175±396 | 0.85±0.43 | 1.26±0.99 |
| **Garen Manvelian et al (2012)**  **‐ fasted condition** | 50 | 1316±577 | - | 1511±389 | 1.92±0.38 | 0.8±0.50 |
| **Garen Manvelian et al (2012)**  **‐ fed condition** | 50 | 951±391 | - | 1375±325 | 2.2±0.59 | 1.74±1.15 |

* Values from this study are presented as range (minimum–maximum), while reference values are presented as mean ± standard deviation

**Supplementary data**

**R code for biomimetic *in vitro* system model development**

*In the following examples, comments appear in blue text while* ***code*** *is shown in* ***black****.*

| *### Load library*  library(dplyr)  library(nlmixr2)  *### Load observed data*  df = …  *### Save model as function*  model = function() {  ini({  *### Set the initial value for each parameters*  *### Weibull distribution parameters for parent drug and metabolite*  lvmax_p <- log(0.0631)  lvmax_m <- log(0.03)  alpha <- fix(1.565)  beta <- fix(-3.3)  lmeta <- log(4.71e-06) *# cellular metabolic clearance (mL/min/cell)*  lportion <- log(0.0155) *# metabolic conversion fraction*    V2 <- fix(1) *# volume of insert = 1 mL*    sf <- 0.643 *# scaling factor for cell counts*  eta.meta ~ fix(0) *# fixed variability as 0 in model development step*  *# eta.meta can be changed in simulation steps*    add.err <- fix(0) *# 0% for random variability of parent drug*  add.err_M <- fix(0) *# 0% for random variability of metabolite*  })  model({  *### Change model parameters from log scale to linear scale*  vmax_p = exp(lvmax_p)  vmax_m = exp(lvmax_m)  portion = expit(lportion)  *### Designate rate constants for parent drug and metabolite with Weibull distribution equation*  wb = (1 - exp(-((time / alpha)^beta)))  *### Determine metabolic clearance by incorporating the influence of cell count.*  *### Cell count information is included in the input data.*  *### Alternatively, this information can be directly entered in the initialization section.*  CL_meta = exp(lmeta + sf*log(Cell_Count) + eta.meta)  elimA2 = CL_meta * A2  *### Save flow rates for each compartment*  flowA = vmax_p * (A1 - A2) * wb # parent flow  flowAm = vmax_m * (A1_Met - A2_Met) * wb # metabolite flow  *### Differential equation for each compartments*  d/dt(A1) = - flowA *### media compartment of parent drug*  d/dt(A2) = flowA - elimA2 *### insert compartment of parent drug*  d/dt(A1_Met) = - flowAm *### media compartment of parent drug*  d/dt(A2_Met) = flowAm + elimA2 * portion * (312.1/296.148) *### insert compartment of metabolite*    *### Calculate concentration for parent drug and metabolite*  *### Specifies concentration for observed values in linear scale, or log-transformed concentration for observed values in log scale. ### C2 is for concentration of parent drug in insert, and C2_Met is for the metabolite*  C2 <- log(A2/V2)  C2_Met <- log(A2_Met/V2)    C2 ~ add(add.err) \| A2  C2_Met ~ add(add.err_M) \| A2_Met  })  }  *### Comfile the model*  mod <- nlmixr2(model)  *### Conduct estimation*  fit <- nlmixr2(mod, df, "focei", control = foceiControl(seed = 1234)) |
| --- |
